# Prevalence and influencing factors of cognitive frailty in patients with chronic heart failure: a meta-analysis

**DOI:** 10.3389/fcvm.2026.1819286

**Published:** 2026-07-20

**Authors:** Meiwan Zhang, Chen Zhou, Min Hu, Chi Tian, Jiayue Zeng

**Affiliations:** 1 School of Nursing, Jiangxi Medical College, Nanchang University, Nanchang, China; 2 Department of Physiology, Basic Medical College, Nanchang University, Nanchang, China; 3 Department of Nursing, The Second Affiliated Hospital of Nanchang University, Nanchang, China; 4 Nanchang University Second Affiliated Hospital, Nanchang, China

**Keywords:** chronic heart failure patients, cognitive frailty, influencing factors, meta-analysis, prevalence

## Abstract

**Objective:**

To systematically investigate the prevalence and influencing factors of cognitive frailty in patients with chronic heart failure, and to provide evidence-based support for subsequent clinical intervention development.

**Methods:**

We retrieved relevant studies focusing on the prevalence and influencing factors of cognitive frailty in patients with chronic heart failure from Embase, Cochrane Library, PubMed, Web of Science, CINAHL, SinoMed, CNKI, VIP, and Wanfang databases. The search period covered from the inception of each database to December 2025. Two researchers independently conducted study screening, data extraction, and methodological quality assessment. Meta-analysis was performed using Stata 15.0 software.

**Results:**

Fourteen studies involving 5,127 patients with chronic heart failure were included, among which 1,875 cases of cognitive frailty were reported, and 30 risk factors were identified. Meta-analysis showed that the pooled prevalence of cognitive impairment in patients with chronic heart failure was 39.3% (95% CI: 31.0%–47.7%). Further analysis identified age, age ≥ 80 years, New York Heart Association (NYHA) classification, comorbidity burden, polypharmacy, malnutrition, and depression as significant risk factors for cognitive impairment in this population. Higher educational attainment, engaging in physical exercise ≥ 3 times per week, and regular participation in intellectual activities were confirmed as protective factors against cognitive impairment in patients with chronic heart failure.

**Conclusion:**

Current evidence confirms that cognitive impairment is highly prevalent among patients with chronic heart failure, and its occurrence is influenced by multiple modifiable and non-modifiable risk factors. Therefore, clinical healthcare providers should strengthen routine cognitive function screening for this population, and implement targeted interventions based on the identified risk factors to delay the onset of cognitive impairment and improve overall patient prognosis.

**Systematic Review Registration:**

https://www.crd.york.ac.uk/PROSPERO/view/CRD420251247352, PROSPERO CRD420251247352.

## 1 Introduction

Chronic heart failure (CHF) represents the end-stage progression of multiple cardiovascular diseases. Its prevalence, mortality, and readmission rates remain consistently high, rendering it one of the most critical challenges in global public health (1, 2). Current epidemiological estimates indicate that approximately 64 million individuals worldwide are living with CHF, imposing a substantial socioeconomic burden and straining the capacity of global healthcare systems (3, 4). CHF patients frequently present with multiple comorbidities. Among these, cognitive frailty (CF)—a syndrome characterized by concurrent physical and cognitive impairment (excluding dementia subtypes such as Alzheimer's disease) (5)—has emerged as a prominent research priority at the intersection of cardiology, geriatrics, and public health in recent years. CF is associated with elevated risks of adverse health outcomes in affected patients, including higher mortality and readmission rates, poorer quality of life, and reduced treatment adherence (6). CHF patients with cognitive frailty have a 1.55-fold higher risk of all-cause mortality within one year compared to those with normal cognitive function (7). Therefore, elucidating the prevalence and modifiable influencing factors of cognitive frailty in CHF populations is critical for informing population-level intervention strategies and advancing patient-centered health management frameworks. Currently, cross-sectional studies investigating cognitive impairment in CHF patients globally demonstrate substantial heterogeneity in reported findings, largely attributable to methodological limitations such as small sample sizes, high population heterogeneity, and inconsistent assessment tools. Accordingly, this study conducts a systematic meta-analysis of peer-reviewed research on cognitive impairment in CHF populations to synthesize existing evidence and clarify its pooled prevalence estimates and key influencing factors. This will provide evidence-based guidance for early identification of high-risk populations in clinical settings, community-based early screening, and implementation of targeted preventive interventions.

## 2 Materials and methods

### 2.1 Study design

This systematic review and meta-analysis was performed in compliance with the Preferred Reporting Items for Systematic Reviews and Meta-Analyses (PRISMA) 2020 guidelines. The study protocol has been prospectively registered in the International Prospective Register of Systematic Reviews (PROSPERO) under registration number CRD420251247352.

### 2.2 Search strategy

A systematic literature search was conducted across nine major academic databases: Web of Science, PubMed, Cochrane Library, Embase, CINAHL, China National Knowledge Infrastructure (CNKI), VIP Database for Chinese Technical Periodicals, China Biomedical Literature Database (SinoMed), and Wanfang Data Knowledge Service Platform. The search timeframe covered from the inception of each database to December 2025. No language restrictions were applied. An example of the PubMed search strategy is presented below:[“cognitive frailty”[Title/Abstract] OR “Cognitive Dysfunction”[MeSH Terms]] AND (“chronic heart failure”[Title/Abstract] OR “Heart Diseases”[MeSH Terms] OR “Heart Failure”[MeSH Terms] OR “CHF”[Title/Abstract])Search strategies for the other databases were adjusted accordingly based on their respective syntax rules. The complete search strategies for Chinese databases are provided in the (Supplementary Table S1).

### 2.3 Inclusion and exclusion criteria

#### 2.3.1 Inclusion criteria

(1) Study population: Patients with a clinically confirmed diagnosis of chronic heart failure, regardless of age, gender, or heart failure etiology; (2) Study design: Cross-sectional studies, cohort studies, and case-control studies; (3) Outcome measures: Studies reporting the prevalence of cognitive frailty and/or its associated risk factors, with available data for extracting odds ratios (OR) and corresponding 95% confidence intervals (CI); (4) Diagnostic definition: Cognitive frailty was defined as the concurrent presence of physical frailty and cognitive impairment, with cases of Alzheimer's disease and other forms of dementia explicitly excluded; (5) Language restriction: Studies published in English or Chinese were eligible for inclusion.

#### 2.3.2 Exclusion criteria

(1) Review articles, conference abstracts, duplicate publications, or studies without accessible full-text; (2) Studies assessed as being of low methodological quality based on the adopted quality appraisal tool.

### 2.4 Study selection and data extraction

After preliminary retrieval, EndNote X9 software was used to manage all retrieved literature records, and duplicate entries were removed first. Two researchers with specialized training in evidence-based medicine independently completed literature screening and data extraction. All discrepancies during the screening and extraction process were resolved through discussion with a third senior researcher to reach a final consensus. Extracted data were recorded in a standardized pre-designed Microsoft Excel spreadsheet, including the following items: first author, publication year, study region, sample size, number of participants with cognitive frailty, prevalence of cognitive frailty, assessment tools for cognitive frailty and physical frailty, and reported influencing factors for cognitive frailty.

### 2.5 Quality assessment

Methodological quality assessment of included studies was independently conducted by two researchers. For cross-sectional studies, the evaluation criteria recommended by the Agency for Healthcare Research and Quality (AHRQ) were adopted (8). This tool consists of 11 items with a total possible score of 11 points; studies scoring 0–3 points were categorized as low quality, 4–7 points as moderate quality, and ≥8 points as high quality. For cohort studies, the Newcastle-Ottawa Scale (NOS) was used for quality appraisal (9). This scale has a total possible score of 9 points, with scores of 1–3 indicating low quality, 4–6 indicating moderate quality, and 7–9 indicating high quality. Any discrepancies arising during the quality assessment process were resolved via consultation with a third senior researcher to reach a final consensus.

### 2.6 Statistical analysis

Data analysis was performed using Stata 15.0 statistical software. Effect sizes were expressed as pooled rates and 95% confidence intervals (95% CI). Influencing factors were described using odds ratios (OR) and 95% CI. Heterogeneity among included studies was assessed using Cochran's *Q* test and *I*^2^ test. If *I*^2^ < 50% and *P* ≥ 0.1, indicating low heterogeneity, a fixed-effects model was selected for analysis; otherwise, a random-effects model was used. Subgroup and sensitivity analyses were conducted to explore sources of heterogeneity. Egger's test assessed publication bias for studies with ≥10 included articles; *P* > 0.05 suggested low likelihood of publication bias. *P* < 0.05 was considered statistically significant.

## 3 Results

### 3.1 Literature screening process and results

**Figure 1:**
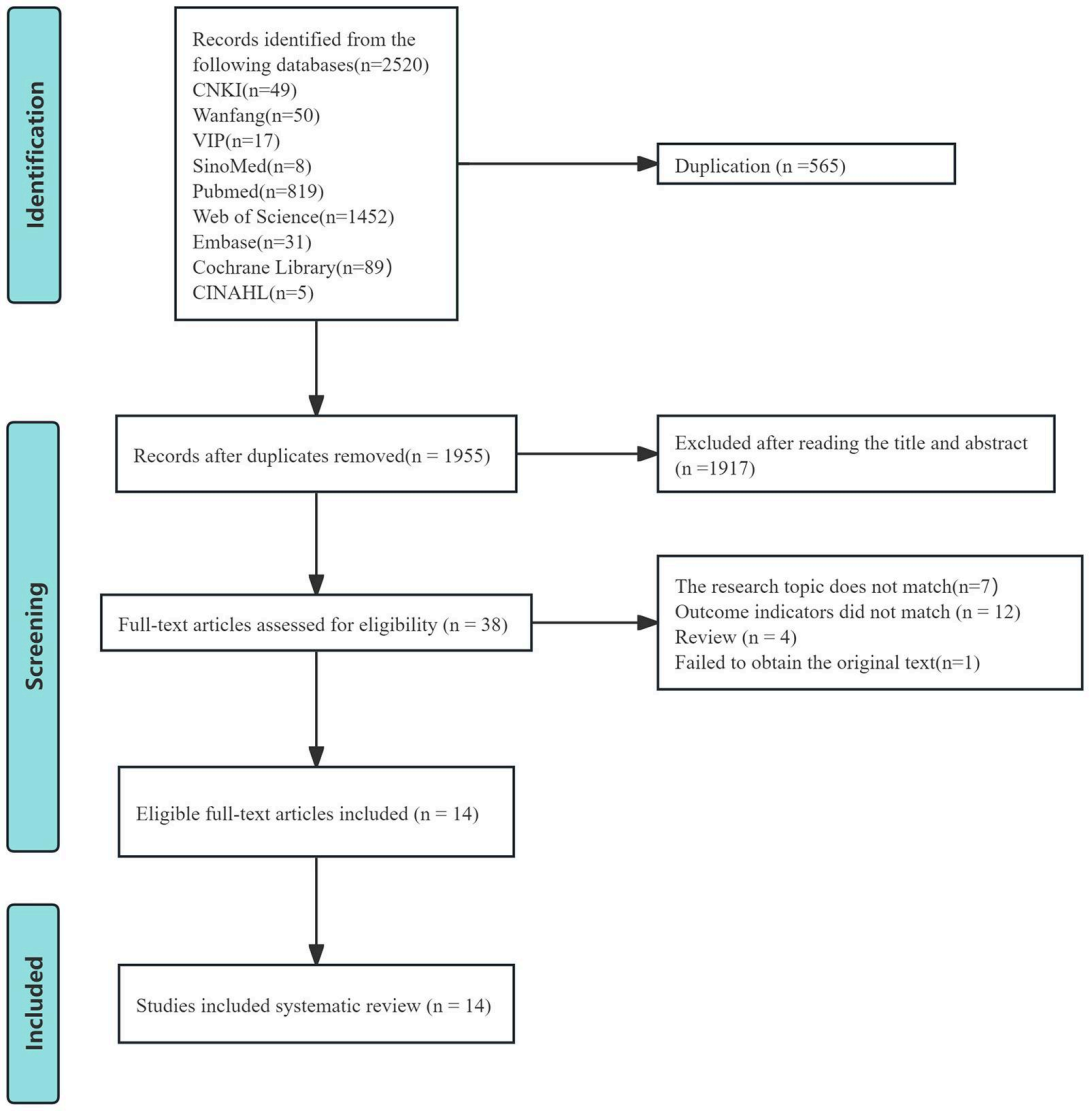
Flow diagram of study selection.

The initial search yielded 2,520 articles. Using EndNote 20 software, 565 duplicate records were removed. After further review of titles and abstracts, 1,917 articles failing to meet inclusion criteria were excluded. Following full-text review, 24 additional articles were excluded for non-compliance with inclusion criteria. Ultimately, 14 articles were included—7 in Chinese (10–16) and 7 in English (7, 17–22). The specific screening process is illustrated in Figure 1.

### 3.2 Study characteristics and quality assessment

This study included 14 publications involving 5,127 patients with CHF, among whom 1,875 developed cognitive impairment. The prevalence of cognitive impairment ranged widely from 19.71% to 75.33%. A total of 30 influencing factors were identified. According to the AHRQ and NOS evaluation criteria, all included studies were of moderate or high quality. The basic characteristics of the included literature and the results of the quality assessment are presented in Table 1.

**Table 1:** Characteristics of the studies included in the meta-­analysis.

Author	Publicati-on Year	Country	Age (years)	*N*	Number of patients with CF	Prevalence of CF (%)	Cognitive frailty criteria		Risk factor	Quality
							Frailty	Cognitive function assessment		
Jha et al. (21)	2016	Australia	≥18	156	62	39.74	FP	MoCA	—	9/9
Yamamoto et al. (7)	2022	Japan	≥65	1,215	279	22.96	FP	Mini-Cog©	—	9/9
Seo et al. (22)	2022	Korea	≥65	168	58	34.52	FS	MMSE	10, 15	7/11
Jiang et al. (10)	2023	China	≥60	261	90	34.48	FP	MoCA	1, 3, 10, 12, 15, 17, 19, 26, 27	8/11
Cui et al. (11)	2024	China	≥18	247	111	44.94	FP	MoCA	—	6/11
Luo et al. (12)	2024	China	≥60	313	96	30.67	FP	MMSE	1, 3, 9, 10, 15, 17, 18, 21	6/11
Wang et al. (13)	2024	China	≥65	124	36	29.03	FP	MMSE	1, 24, 28	6/11
Liu et al. (14)	2024	China	≥60	300	226	75.33	FS	MoCA	1, 3, 12, 14	6/11
Xu et al. (18)	2024	China	≥18	271	134	49.45	FS	MoCA	11, 24, 26, 27	6/11
Guo et al. (17)	2024	China	≥60	279	148	53.05	FS	MMSE	1, 2, 3, 7, 16, 23, 26, 29, 30	5/11
Chen et al. (15)	2024	China	≥60	607	293	48.27	FP	MoCA	1, 3, 4, 5, 9, 23, 24, 26, 29	6/11
Jiang et al. (16)	2025	China	≥60	330	124	37.58	FP	MoCA	1, 3, 9, 12, 15, 17, 26	7/11
Gou et al. (19)	2025	China	≥60	421	83	19.71	FS	MMSE	1, 6, 8, 15, 17, 22, 26	7/11
Li et al. (20)	2025	China	≥60	435	135	31.03	FS	MMSE	1, 9, 13, 15, 17	6/11

FP, frailty phenotype; FS, frailty scale; Mini-Cog©, a cognitive assessment tool combining three recall tests and a clock-drawing test, with testing methods based on its official website (https://mini-cog.com); MoCA, montreal cognitive assessment MMSE, mini mental state examination (1) Age; (2) Gender; (3) Educational attainment; (4) Living alone; (5) Marital status; (6) Economic status; (7) Rural residence; (8) Alcohol consumption; (9) Physical exercise; (10). Sleep; (11). Blood pressure level; (12) Engagement in intellectual activities; (13) Activities of daily living (ADL) ability; (14) Loneliness; (15) Depression; (16) Anxiety; (17) NYHA classification; (18) Heart failure duration; (19) LVEF score; (20) NT-proBNP; (21) Cardiac stent placement; (22) Length of hospital stay; (23) Polypharmacy; (24) BMI; (25) Grip strength; (26) Nutritional status; (27) Level of social support; (28) History of cerebral infarction; (29) Comorbidity; (30) Complications; — indicates absence of this variable.

### 3.3 Results of meta-analysis on the prevalence of cognitive frailty in CHF patients

#### 3.3.1 Prevalence of cognitive frailty

**Figure 2:**
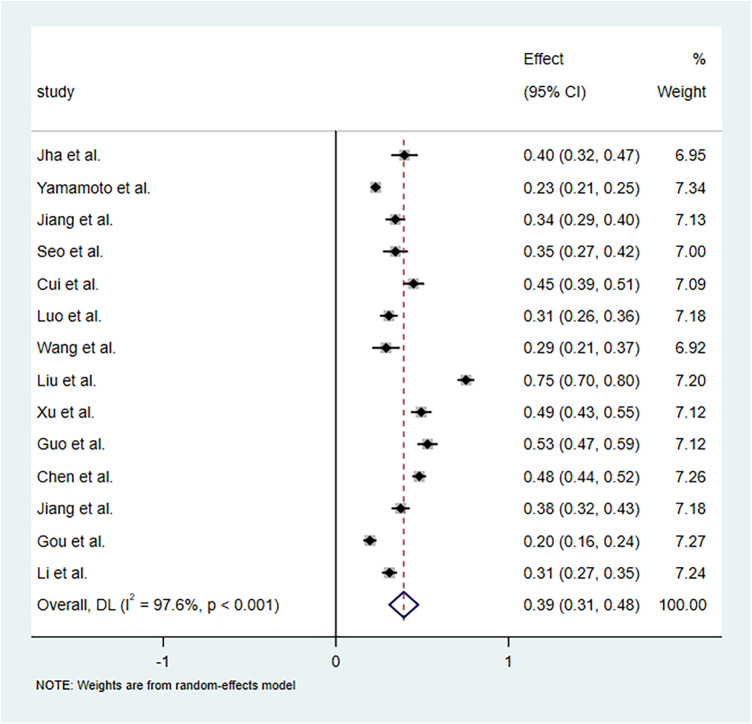
Forest plot of the prevalence of cognitive frailty in CHF patients.

A meta-analysis of prevalence rates across the 14 included studies revealed significant heterogeneity $I^{2}=97.6\text{\%},P<0.001$. Therefore, a random-effects model was employed for the pooled analysis. Results indicated that the prevalence of cognitive frailty among CHF patients was 39.3% (95% CI = 31.0%–47.7%), as shown in Figure 2.

#### 3.3.2 Subgroup analysis

Subgroup analyses were performed based on cognitive frailty assessment tools, physical frailty assessment tools, cognitive function assessment tools, study region, study publication year, gender, and nightly sleep duration. The results were as follows: (1) Subgroup analysis by cognitive frailty assessment tool combination revealed significant differences in pooled cognitive frailty prevalence across different measurement pairs: the FP + Mini-Cog© combination had the lowest prevalence at 23.0%, while the FS + MoCA combination had the highest prevalence at 55.0%; (2) Subgroup analysis by study region showed that the pooled prevalence of cognitive frailty in CHF patients was comparable between East Asia (39.3%) and Oceania (39.7%); (3) Subgroup analysis by publication year indicated that the pooled prevalence of cognitive frailty in studies published after 2023 (41.9%) was higher than that in studies published before 2023 (32.5%); (4) Gender-based subgroup analysis found that female CHF patients had a higher pooled prevalence of cognitive frailty (39.6%) than male patients (32.1%); (5) Subgroup analysis by nightly sleep duration demonstrated that CHF patients with nightly sleep duration ≥6 h had a significantly lower pooled prevalence of cognitive frailty (28.6%) than those with nightly sleep duration <6 h (39.6%). Detailed results are presented in Table 2. Notably, the FP + Mini-Cog© subgroup contained only one study, and its pooled prevalence was merely for descriptive reference. Stratified data based on LVEF, heart failure etiology and research setting were unavailable from primary studies, so further subgroup decomposition for heterogeneity exploration could not be conducted.

**Table 2:** Subgroup analysis of the prevalence of cognitive frailty in CHF patients.

Subgroup analysis	*N*	Heterogeneity		Effect model	Prevalence (%)	95% CI
		*I*^2^ (%)	*p*			
Cognitive frailty assessment tool						
FP + Mini-Cog©	1 (7)	–	–		23.0	20.6–25.3
FP + MoCA	4 (10, 11, 15, 16)	84.6	<0.001	random-effect model	41.4	34.8–48.1
FS + MMSE	3 (17, 19, 20)	97.7	<0.001	random-effect model	34.5	16.9–52.0
FP + MMSE	3 (12, 13, 22)	0	0.566	fixed-effect model	31.3	27.6–35.0
FS + MoCA	3 (18, 20, 21)	97.7	<0.001	random-effect model	55.0	33.3–76.7
Frailty assessment tool						
FP	8 (7, 10–13, 15, 16, 22)	95.1	<0.001	random-effect model	35.3	27.6–43.0
FS	6 (17–21)	98.6	<0.001	random-effect model	44.7	27.1–62.3
Cognitive function assessment tool						
Mini-Cog©	1 (7)	–	–		23.0	20.6–25.3
MoCA	7 (10, 11, 15, 16, 18, 20, 21)	96.3	<0.001	random-effect model	47.2	36.5–57.9
MMSE	6 (12, 13, 17, 19, 20, 22)	94.4	<0.001	random-effect model	32.9	23.7–42.1
Region						
Oceania Region Group	1 (21)				39.7	32.1–47.4
East Asian Region	13 (7, 10–13, 15–17, 19, 20, 22)	97.8	<0.001	random-effect model	39.3	30.5–48.1
Study year						
Before 2023	4 (7, 10, 21, 22)	90.7	<0.001	random-effect model	32.5	23.8–41.2
After 2023	10 (11–20)	97.7	<0.001	random-effect model	41.9	31.3–52.5
Sex						
Male	12 (7, 10–13, 15, 16, 18–22)	93.2	<0.001	random-effect model	32.1	25.2–38.9
Female	12 (7, 10–13, 15, 16, 18–22)	88.6	<0.001	random-effect model	39.6	33.1–46.0
Sleep duration (hours)						
<6 h	3 (10, 12, 22)	63.3	0.066	random-effect model	39.6	29.7–49.5
≥6 h	3 (10, 12, 22)	76.7	0.014	random-effect model	28.6	20.1–37.1

### 3.4 Meta-analysis results on factors influencing cognitive frailty in CHF patients

Associations between 10 potential influencing factors and cognitive frailty were synthesized, with detailed statistical parameters shown in Table 3. Only two original studies provided fully multivariate-adjusted effect sizes eligible for pooled synthesis, and publication bias tests could not be performed due to insufficient study quantity. (1) Socio-demographic factors: Older age was identified as a significant independent risk factor for cognitive frailty among patients with CHF: per 1-year increment in age was associated with a 13% higher risk of cognitive frailty (pooled OR = 1.130, 95% CI: 1.111–1.150). Subgroup analysis further revealed a particularly pronounced risk elevation in patients aged ≥80 years (pooled OR = 5.711, 95% CI: 3.179–10.262), whereas no statistically significant association was detected in the subgroup of patients aged 70–79 years. Regarding educational level, higher educational attainment (college degree or above) acted as a protective factor against cognitive frailty (pooled OR = 0.182, 95% CI: 0.099–0.337), while no significant correlation was observed between primary education level and cognitive frailty risk in this population. (2) Lifestyle factors: Weekly physical exercise ≥3 times (pooled OR = 0.164, 95% CI: 0.100–0.269) and regular participation in intellectual activities (pooled OR = 0.177, 95% CI: 0.038–0.831) were identified as protective factors against cognitive frailty in CHF patients. No statistically significant association was detected between BMI and cognitive frailty risk in this population. (3) Disease-related factors:Elevated NYHA classification (pooled OR = 2.566, 95% CI: 1.687–3.904), polypharmacy (pooled OR = 2.278, 95% CI: 1.690–3.070), multimorbidity (pooled OR = 1.919, 95% CI: 1.425–2.583), malnutrition (pooled OR = 2.156, 95%CI: 1.686–2.756), and depression (pooled OR = 4.372, 95% CI: 3.218–5.940) were all significantly associated with increased risk of cognitive frailty in CHF patients.

**Table 3:** Meta-analysis of factors affecting cognitive frailty in CHF patients .

Risk factor	*N*	Heterogeneity		Meta-analysis	
		*I*^2^ (%)	*P*	OR [95% CI]	*P*
Age	4 (13, 16, 17, 20)	0	0.65	1.130 (1.111–1.150)	<0.001
70–79 years	2 (14, 15)	87.8	0.004	0.991 (0.308–3.190)	0.988
≥80 years	3 (12, 15, 19)	0	0.664	5.711 (3.179–10.262)	<0.001
Educational level					
Primary School	2 (14, 15)	92.9	<0.001	1.159 (0.164–8.200)	0.882
College and above	3 (10, 12, 17)	29.9	0.24	0.182 (0.099–0.337)	<0.001
Exercise frequency of more than 3 times a week	3 (12, 15, 16)	0	0.804	0.164 (0.100–0.269)	<0.001
Participation in intellectual activities	2 (10, 16)	84.2	0.012	0.177 (0.038–0.831)	0.028
BMI	3 (13, 15, 18)	90	<0.001	0.907 (0.716–1.148)	0.416
NYHA	2 (16, 20)	0	0.439	2.566 (1.687–3.904)	<0.001
Polypharmacy	2 (15, 17)	0	0.947	2.278 (1.690–3.070)	<0.001
Multimorbidity	2 (15, 17)	0	0.955	1.919 (1.425–2.583)	<0.001
Nutritional status					
Malnutrition risk	4 (10, 16, 17, 19)	30.9	0.216	2.156 (1.686–2.756)	<0.001
Nutritional status score	2 (15, 18)	0	0.812	0.824 (0.728–0.933)	0.002
Depression	6 (10, 12, 16, 19, 20, 22)	45.1	0.105	4.372 (3.218–5.940)	<0.001

### 3.5 Sensitivity analysis

**Figure 3:**
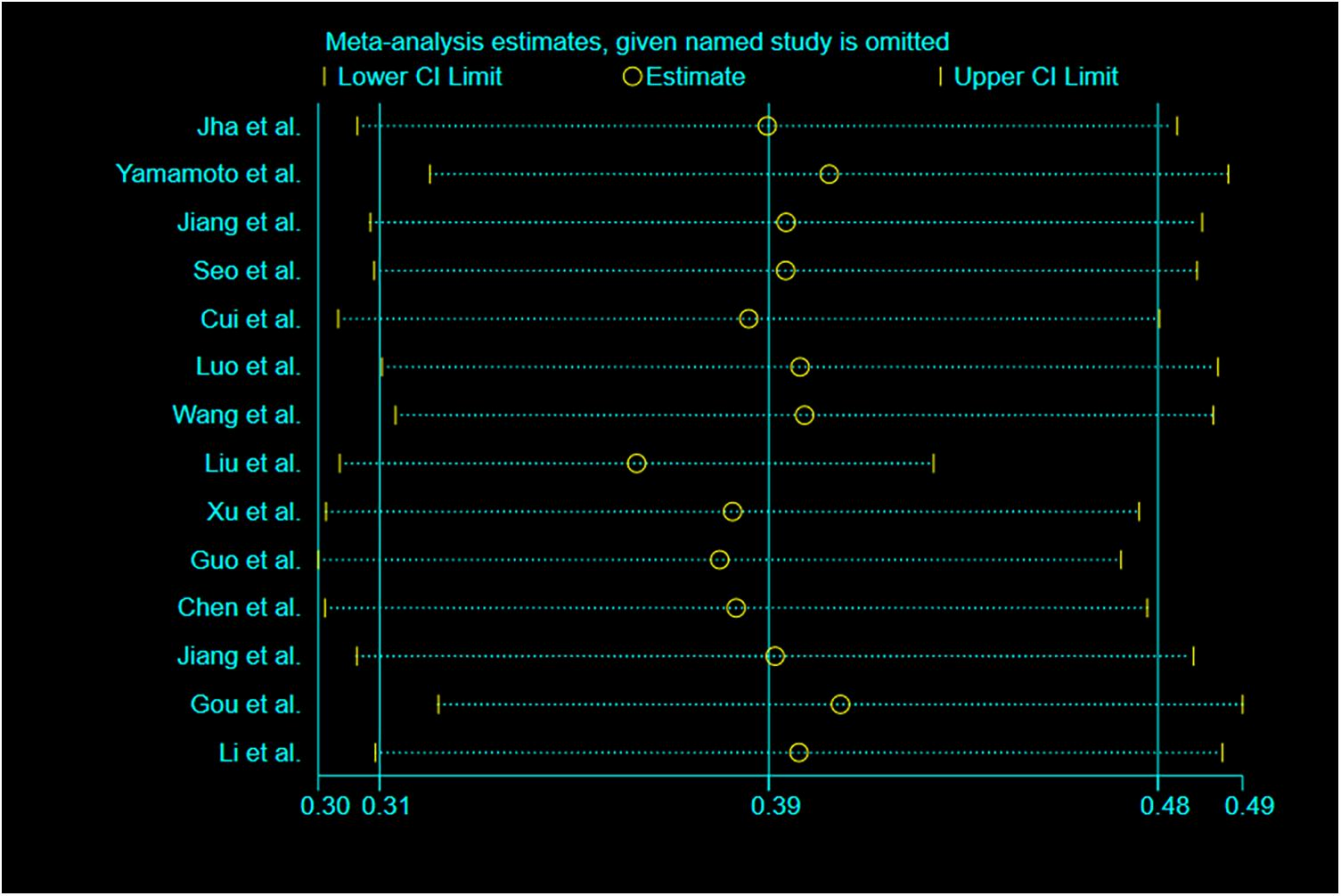
Sensitivity analysis of the overall prevalence rate of cognitive frailty in patients with heart failure.

Sensitivity analysis was performed via stepwise elimination of individual studies for outcomes with *I*^2^ > 50% and ≥3 included studies. The pooled prevalence estimate remained stable after sequential exclusion, with no significant alteration in effect size (sensitivity plot shown in Figure 3). For influencing factors, stability was tested by switching between fixed- and random-effects models: except for the 70–79 years subgroup, primary education level and BMI, all other factors showed minimal effect size changes and no reversed effect direction, indicating robust results (detailed in Table 4).

**Table 4:** Sensitivity analysis of factors affecting cognitive frailty in CHF patients.

Risk factor	Fixed effect model OR [95% CI]	Random effect model OR [95% CI]
Age	1.130 (1.111–1.150)	1.130 (1.111–1.150)
70–79 years	1.029 (0.685–1.546)	0.991 (0.308–3.190)
≥80 years	5.711 (3.179–10.262)	5.711 (3.179–10.262)
Educational level		
Primary School	0.917 (0.554–1.518)	1.159 (0.164–8.200)
College and above	0.182 (0.099–0.337)	0.170 (0.079–0.366)
Exercise frequency of more than 3 times a week	0.164 (0.100–0.269)	0.164 (0.100–0.269)
Participation in intellectual activities	0.233 (0.133–0.409)	0.177 (0.038–0.831)
BMI	0.968 (0.903–1.038)	0.907 (0.716–1.148)
NYHA	2.566 (1.687–3.904)	2.566 (1.687–3.904)
Polypharmacy	2.278 (1.690–3.070)	2.278 (1.690–3.070)
Multimorbidity	1.919 (1.425–2.583)	1.919 (1.425–2.583)
Nutritional status		
Malnutrition risk	2.156 (1.686–2.756)	2.307 (1.675–3.178)
Nutritional status score	0.824 (0.728–0.933)	0.824 (0.728–0.933)
Depression	4.372 (3.218–5.940)	4.457 (2.872–6.918)

### 3.6 Publication bias

Publication bias analysis was conducted using Egger's test for studies with ≥10 included papers. Results showed: However, the Egger's test *P*-values for male and female prevalence rates were both <0.05, indicating significant publication bias. After further adjustment using trimming methods, the *P*-value remained <0.05, and the pooled results showed relative stability.

## 4 Discussion

### 4.1 Prevalence of cognitive frailty in CHF patients

This study included 14 research articles. Meta-analysis results showed that the prevalence of cognitive impairment among CHF patients was 39.3% (95% CI: 31.0–47.7%), significantly higher than the prevalence of cognitive frailty in the elderly reported by Wang et al. (23). A classic cross-sectional population study targeting older adults verified that congestive heart failure is an independent risk factor for cognitive dysfunction, with markedly elevated risk of cognitive abnormalities in patients with CHF (24). This discrepancy may be related to differences in the study populations: this study focused on patients with chronic cardiovascular disease (CHF), whereas Wang et al.'s study included general elderly individuals without specific chronic diseases. Compared to other chronic disease populations, the prevalence of cognitive impairment among CHF patients in this study was higher than that observed in patients undergoing maintenance hemodialysis (25%) (25) and elderly diabetic patients (24.3%) (26), suggesting a higher risk of cognitive impairment in CHF patients. Therefore, clinical practice should prioritize early identification and systematic screening for cognitive frailty in CHF patients. Effective interventions such as cognitive training and nutritional support should be promptly implemented for screen-positive individuals to delay cognitive frailty progression and improve patient outcomes.

This study conducted subgroup analyses of included studies across five dimensions: assessment tools, study region, publication period, gender, and sleep duration. (1) Assessment Tools: The combined FRAIL frailty scale and MoCA yielded the highest pooled cognitive frailty prevalence in CHF patients. Subgroup analyses showed prevalence was significantly higher in groups using either FRAIL or MoCA alone vs. other screening tools. The FRAIL scale covers five domains (fatigue, resistance, ambulation, illness burden, weight loss) and enables rapid identification of high-risk CHF patients by integrating subjective symptoms, comorbidities, and daily function assessments (27). The MoCA assesses attention, memory, visuospatial function, and language, with high sensitivity for early cognitive impairment in CHF patients (28). Their combined use has good sensitivity and predictive efficacy, fits CHF clinical characteristics, and supports early identification and intervention. Combined application of the two scales realizes multidimensional evaluation covering physical and cognitive function. As documented in prior research, multi-domain frailty assessment covering physical, psychological and social dimensions enables more accurate risk stratification among elderly outpatients with heart failure (29). Incorporating this integrated evaluation mode into daily clinical work facilitates timely screening of high-risk CHF individuals. (2) Study Region: Cognitive frailty prevalence in Oceania was slightly higher than in East Asia, inconsistent with Cheng et al.'s (30) finding of higher prevalence in Asian populations. This discrepancy likely reflects the small number of Oceania studies included, leading to limited sample representativeness. Future multicenter studies should account for regional differences in healthcare resources and economic conditions to generate more robust prevalence estimates. (3) Time: Studies published 2023 onwards reported higher prevalence than pre−2023 studies, potentially due to limited prior research on this topic. (4) Gender: Prevalence was higher in female vs. male CHF patients, consistent with Xie et al. (31). This may be explained by lower female muscle mass, plus postmenopausal estrogen fluctuations and altered inflammatory cytokine profiles, which exacerbate cognitive damage (32). (5) Sleep Duration: Patients with <6 h of nightly sleep had higher cognitive frailty risk, consistent with prior research (33). This may be mediated by sleep deprivation-induced cortisol elevation and circadian disruption, which drive cognitive decline (34).

### 4.2 Factors associated with cognitive frailty in CHF patients

#### 4.2.1 Sociodemographic characteristics

Age is identified as an independent risk factor for cognitive frailty in CHF patients, with risk increasing significantly with advancing age, consistent with prior published evidence (30, 31, 35). Our study further demonstrates that the prevalence of cognitive frailty in patients aged ≥80 years is 5-fold higher than in those aged <80 years. Physiological declines associated with aging include impaired audiovisual function and significant skeletal muscle mass loss (36), alongside accelerated atrophy of the hippocampus and cerebral cortex, which drives progressive cognitive impairment and contributes to cognitive frailty progression (36, 37). Furthermore, reduced social participation and lower resilience to external stimuli among older adults impair information processing efficiency and physical activity capacity, further elevating cognitive frailty risk (10). Clinical practitioners should therefore prioritize cognitive frailty monitoring in elderly CHF populations, with a focus on early screening and preventive strategies. Higher educational attainment is a protective factor for cognitive frailty in CHF patients, consistent with the findings of Xie et al. (31). Individuals with higher education levels typically engage in long-term cognitive activity, which sustains neuronal activity, preserves synaptic plasticity and cognitive reserve, and strengthens the brain's compensatory capacity against aging-related damage (38). This population also demonstrates greater initiative in acquiring disease management knowledge and has stronger self-health management skills, which further reduce cognitive frailty risk (39). Accordingly, healthcare providers should develop personalized intervention plans tailored to patients' educational backgrounds, and encourage regular daily cognitive stimulation practices to delay cognitive decline progression.

#### 4.2.2 Lifestyle factors

This study identified that exercising three or more times per week acts as a protective factor against cognitive frailty in patients with CHF, consistent with findings reported by Noji T et al. (40). Regular physical activity improves cerebral perfusion, attenuates neuronal damage, suppresses pro-inflammatory responses, and reduces the risk of cognitive decline (41). From a public health practice perspective, community healthcare providers can develop standardized, tailored exercise prescriptions for CHF patients based on their clinical stage and exercise tolerance, supporting appropriate increases in exercise frequency to delay cognitive decline onset at the population level. Furthermore, regular participation in intellectual activities is also a protective factor against cognitive decline in CHF patients, which aligns with the findings of Fang et al. (42). Engagement in intellectual activities provides sustained cognitive stimulation, enhances neuroplasticity, and effectively improves cognitive reserve (43). For public health intervention, primary care providers can encourage CHF patients to actively participate in diverse cognitive training activities and access novel cognitive experiences, to promote neural activity and delay the onset of cognitive decline across the CHF patient population.

#### 4.2.3 Disease-related factors

This study identified NYHA classification as an independent risk factor for cognitive frailty in CHF patients: worse NYHA class is associated with significantly higher cognitive frailty risk, consistent with prior evidence (44, 45). Cardiac dysfunction in CHF patients causes multi-organ hypoperfusion, particularly insufficient cerebral blood flow and neuronal ischemia-hypoxia injury, which impairs cognitive function (46). From a public health perspective, standardized cardiac rehabilitation should be integrated into community CHF management, with primary care providers delivering tailored home-based guidance for stable patients to improve cardiac function at the population level. Additionally, multimorbidity and polypharmacy are also independent risk factors for cognitive frailty in CHF patients. Pathological interactions between coexisting diseases exacerbate cerebral ischemia-hypoxia, increasing cognitive decline risk (47). Polypharmacy is associated with elevated pro-inflammatory factors, which cross the blood-brain barrier to trigger neuroinflammation and neuronal dysfunction (48). These findings highlight the need to strengthen multimorbidity management and medication safety monitoring for CHF populations: optimizing regimens and reducing inappropriate polypharmacy can lower cognitive frailty risk and reduce the overall public health burden of CHF complications.

This study also identified malnutrition as a risk factor for cognitive frailty in CHF patients, consistent with previous evidence (30, 49). Malnourished CHF patients often present with significant skeletal muscle loss, higher fracture and fall risk, and increased physical frailty vulnerability (50). Furthermore, the “reverse metabolic syndrome” or “catabolic syndrome” described by Curcio et al. (51) in elderly populations—characterized by low BMI, low blood pressure, and low cholesterol paradoxically associated with poorer outcomes—may further exacerbate the vulnerability of malnourished CHF patients to cognitive frailty. This catabolic state, closely linked to sarcopenia and physical frailty, may simultaneously accelerate cognitive decline through chronic inflammation and metabolic dysregulation (51). In addition, deficiencies of multiple vitamins and trace elements in malnourished individuals can directly impair cognitive function (52). Public health interventions should incorporate nutritional screening into routine health management for CHF patients, guiding them to develop sound dietary habits, ensure timely nutritional supplementation, enhance their nutritional literacy, and reduce the risk of cognitive decline. Furthermore, this study confirmed depression as an independent risk factor for cognitive frailty in CHF patients, aligning with prior studies (35, 53). Depression, cognitive impairment, and frailty share common risk factors and overlapping pathophysiological pathways (54). Long-term disease burden in CHF patients can induce depressive symptoms, which in turn lead to social isolation and reduced physical activity, accelerating skeletal muscle loss, declining physical function, and increasing physical frailty risk (54). For public health intervention, these results highlight the need to incorporate routine depression screening into CHF management, with early identification of negative emotions and timely psychological support to improve cognitive flexibility, delay cognitive decline, and optimize patient outcomes. Notably, sacubitril/valsartan has been shown to significantly alleviate depressive symptoms in patients with advanced heart failure, with the proportion of patients with depression decreasing from 59.5% to 21.6% after one year of treatment (55). These findings suggest that optimizing heart failure treatment may confer simultaneous benefits for both cardiac and psychological health, underscoring the importance of integrated cardiology-psychiatry management in the chronic heart failure population.

Limitations of this study: First, due to constraints in sample size and data types across the included studies, some influencing factors could only be analyzed descriptively. Second, the use of different cognitive assessment tools across studies may have introduced measurement bias. Third, the inclusion of only Chinese- and English-language literature may have resulted in language bias. Fourth, insufficient data reporting in the original studies precluded subgroup analyses by left ventricular ejection fraction, heart failure etiology, or patient setting; these unexamined clinical characteristics may have contributed to the observed heterogeneity. Fifth, disease duration, cardiac medications and comorbidity severity are potential confounders for cognitive function, yet these key variables were neither reported nor controlled for in the original studies, which may have introduced residual confounding bias. Sixth, uniform data on multidimensional frailty assessment and catabolic syndrome phenotypes recommended by Testa et al. and Curcio et al. were unavailable in primary studies, restricting deeper analysis of the malnutrition-frailty-cognitive decline pathway. Future large-scale, multicenter cohort studies are warranted to more comprehensively evaluate the prevalence and dynamic risk factors of cognitive decline in patients with chronic heart failure.

## 5 Conclusion

In summary, this study demonstrates that the prevalence of cognitive frailty in patients with chronic heart failure (CHF) is 39.3%, representing a relatively high disease burden. Age, educational level, exercise frequency, participation in intellectual activities, NYHA functional class, multimorbidity, polypharmacy, nutritional status, and depressive symptoms are associated factors of cognitive frailty in CHF patients. From a public health practice perspective, standardized cognitive frailty screening should be integrated into routine community-based CHF chronic disease management. Multi-disciplinary teams should conduct regular screening to accurately identify high-risk populations, develop personalized multi-domain intervention strategies, and dynamically adjust intervention plans through regular follow-up, to delay the onset of cognitive frailty, reduce the overall CHF-related disease burden, and improve patient quality of life.

## Data Availability

The original contributions presented in the study are included in the article/Supplementary Material, further inquiries can be directed to the corresponding author.
