# Supplementary material for: Prevalence and influencing factors of cognitive frailty in patients with chronic heart failure: a meta-analysis
Source: Front Cardiovasc Med. 2026 Jul 20;13:1819286. doi: 10.3389/fcvm.2026.1819286 (PMC13429837; doi:10.3389/fcvm.2026.1819286)
Supplement: Supplementary file 1 [file Table1.pdf]

Table S1. Chinese Database Search Strategy

Database	Search Strategy
CNKI	(主题=心力衰竭 OR 主题=心衰 OR 主题=慢性心衰 OR 主题=慢性心力衰竭 OR 主题=心功能不全) AND (主题=认知衰弱 OR 主题=认知障碍 OR 主题=认知功能 OR 主题=认知损害 OR 主题=认知衰退) AND (主题=患病率 OR 主题=发生率 OR 主题=影响因素 OR 主题=危险因素 OR 主题=相关因素)
VIP	(题名或关键词=心力衰竭 OR 题名或关键词=心衰 OR 题名或关键词=慢性心衰) AND (题名或关键词=认知衰弱 OR 题名或关键词=认知障碍 OR 题名或关键词=认知功能) AND (题名或关键词=患病率 OR 题名或关键词=影响因素)
SinoMed	("心力衰竭"[常用字段] OR "慢性心力衰竭"[常用字段] OR "心衰"[常用字段]) AND ("认知衰弱"[常用字段] OR "认知障碍"[常用字段] OR "认知功能"[常用字段]) AND ("患病率"[常用字段] OR "影响因素"[常用字段] OR "危险因素"[常用字段])
Wanfang	(主题=心力衰竭 OR 主题=心衰 OR 主题=慢性心衰) AND (主题=认知衰弱 OR 主题=认知障碍 OR 主题=认知功能) AND (主题=患病率 OR 主题=影响因素 OR 主题=危险因素)
